# Characteristics of Circulating CD4^+^ T Cell Subsets in Patients with *Mycobacterium avium* Complex Pulmonary Disease

**DOI:** 10.3390/jcm9051331

**Published:** 2020-05-03

**Authors:** Sun Ae Han, Yousang Ko, Sung Jae Shin, Byung Woo Jhun

**Affiliations:** 1 Division of Pulmonary and Critical Care Medicine, Department of Medicine, Samsung Medical Center, Sungkyunkwan University School of Medicine, Seoul 06351, Korea; maysunae@naver.com; 2 Division of Pulmonary, Allergy and Critical Care Medicine, Department of Internal Medicine, Kangdong Sacred Heart Hospital, Hallym University College of Medicine, Seoul 05355, Korea; koyus@naver.com; 3 Department of Microbiology, Institute for Immunology and Immunological Diseases, Brain Korea 21 PLUS Project for Medical Science, Yonsei University College of Medicine, Seoul 03722, Korea

**Keywords:** nontuberculous mycobacteria, *Mycobacterium avium* complex, *Mycobacterium avium*, *Mycobacterium intracellulare*, CD4^+^ T cells, immunophenotype

## Abstract

Although prevalence of *Mycobacterium avium* complex pulmonary disease (MAC-PD) is increasing, limited data are available regarding vulnerability to *Mycobacterium avium* complex (MAC) infections. To understand the pathobiology of interaction between MAC and host-immunity, it is important to understand the characteristics for circulating T cells in terms of the immunological phenotype and functional correlates in MAC-PD. We aimed to characterize immunophenotype, cytokine profile, and immune inhibitory receptors of circulating CD4^+^ T cells in MAC-PD patients. We enrolled 71 MAC-PD and 20 control individuals. Flow cytometric analysis was performed to determine T cell subsets and immune checkpoint markers. Ex vivo cytokine productions in response to MAC were determined using enzyme-linked immunosorbent assay. The frequencies of CD4^+^ T cells and CD4^+^IL-17^+^ T cells decreased, while CD4^+^IL-4^+^ T cells and CD4^+^CD25^+^Foxp3^+^ T cells increased in peripheral blood mononuclear cells (PBMCs) of MAC-PD individuals upon MAC stimulation compared with those cells in healthy donor-PBMCs. Additionally, we found increased PD-1, CTLA-4, and TIM-3-expressing T cells in MAC- PD individuals in response to MAC-stimulation, indicating that suppressed T cell-mediated response is associated with the susceptibility to MAC infection. These results may help to explain impaired T cell-mediated responses and pave the way for better strategies to achieve protective immunity against MAC infection.

## 1. Introduction

Nontuberculous mycobacteria (NTM) are ubiquitous organisms that can cause chronic pulmonary disease (PD), and the burden of the disease is rapidly increasing worldwide [1,2,3]. *Mycobacterium avium* complex (MAC), including *M. avium* and *M. intracellulare*, is the major causative organisms of NTM-PD. The risk factors for the development of MAC-PD are believed to be not only existing structural lung disease such as bronchiectasis or post tuberculosis (TB) fibrosis, but also dysregulated host response to MAC infection [4]. However, to date, the basis for vulnerability to MAC-PD from an immunologic perspective has yet to be elucidated.

CD4^+^ T cells have long been known to play an important role in immune containment of mycobacterial infection such as *M. tuberculosis*, proven by increased susceptibility to TB in mice lacking CD4^+^ T cells [5,6,7]. CD4^+^ T cells differentiate into numerous T cell subpopulation, such as Th1, Th2, Th17, and regulatory T cells (Tregs), a process regulated by specific transcription factors [8,9]. Although the IFN-γ/IL-12 axis, representative of Th1, is central to disseminated MAC-PD susceptibility [10,11,12,13,14,15,16], its role in patients confined to MAC-PD is unclear. In addition to Th1 cells, Th17 cells also play a role in establishing protective immunity against mycobacterial infections by secreting IL-17 which leads to recruitment and activation of neutrophils. Data have also shown that the function of IL-17 can help in controlling MAC-PD, as well as TB or other bacteria. Additionally, data have revealed that Tregs are involved in mycobacterial infection. For example, numbers of CD4^+^CD25^+^Foxp3^+^ T cell, subsets of Tregs, are elevated upon TB infection leading to suppression of T-cell mediated IFN-γ production, and IFN-γ knockout mice infected with *M. massiliense* showed progressive pulmonary disease and accumulation of Tregs in the lungs. These traits are indicative of T cell dysfunction in NTM infection and raise the possibility of T cell exhaustion in the chronic phase of MAC infection. T cell dysfunction is mediated by several inhibitory pathways including programmed death-1 (PD-1), cytotoxic T-lymphocyte antigen 4 (CTLA-4), and T cell immunoglobulin and mucin domain-containing-3 (TIM-3) pathways, which are widely known targets in cancer immunotherapy [17]. Several models of chronic viral infections, including chronic human immunodeficiency virus (HIV), hepatitis C virus, and hepatitis B virus, have also been linked to high expression of these inhibitory receptors [18,19,20]. Additionally, these receptors play an important role in T cell dysfunction during chronic mycobacteria infections such as TB [21,22,23]. However, there are only a few reports addressing the role of PD-1 and CTLA-4 pathways, especially in MAC-PD [24,25].

In these contexts, however, there are limited data on the characteristics of circulating CD4^+^ T cell subsets in MAC-PD patients. Therefore, we aimed to characterize T cell immune phenotype and immune inhibitory receptor in MAC-PD patients compared with healthy controls, by investigating levels of cytokines, proportion of T lymphocytes, and expression of immune checkpoint inhibitors, PD-1, CTLA-4, and TIM-3, on T lymphocytes. Our data may partly help to identify vulnerability to developing MAC-PD and targets of further study.

## 2. Materials and Methods

### 2.1. Study Population: Patients and Controls

The study included 71 patients with treatment naïve MAC-PD who had visited Samsung Medical Center (Seoul, South Korea) between April 1, 2016 and July 31, 2017. MAC-PD was diagnosed according to American Thoracic Society/Infectious Diseases Society of America criteria based on clinical, radiological, and microbiological findings [1]. Among the enrolled patients, 41 (57.7%) were identified as having *M. intracellulare* infection, and the remaining 30 (42.3%) were identified as having *M. avium* infection. Sixteen women and four men were recruited as healthy control individuals (*n* = 20), who had no history of medical disease such as TB or NTM infection, malignancy, diabetes, viral infection, ongoing treatment prescribed immunosuppressive agents, or pulmonary disease at the time of participation. We tried to select middle-aged to elderly participants as control individuals, because demographic factors can affect immunologic features. All participants provided written informed consent, and the study was approved by the Institutional Review Board of Samsung Medical Center (IRB No. SMC-2008-09-016). Patients with MAC-PD and healthy control subjects provided blood samples at the time of enrolment.

Radiological type of the enrolled patients was evaluated based on chest high-resolution computed tomography at time of MAC-PD diagnosis. The fibro-cavitary form of MAC-PD was defined by presence of cavities and pleural thickening mainly in the upper lobes. The nodular bronchiectatic form was defined by presence of multifocal bronchiectasis and clusters of small nodules, regardless of the presence of small cavities in lungs [26]. 

### 2.2. Cell Preparation and Activations

Peripheral blood mononuclear cells (PBMCs) were isolated using Ficoll–Hypaque density centrifugation (GE Health Care Life Sciences, Uppsala, Sweden), and then suspended in medium containing RPMI-1640 (Life Technologies, NY, NY, USA), 10% fetal bovine serum, and 1% penicillin-streptomycin (Life Technologies, NY, NY, USA). The cells were cryopreserved and stored until needed.

*M. avium* subspecies (American Type of Culture Collection; ATCC 700898) and *M. intracellulare* subspecies (ATCC 25121) were heated at 80°C for 30 min for inactivation. The heat-killed MAC bacilli were used to stimulate PBMCs for the indicated multiplicity of infection (MOI) at a 1:100 ratio respectively.

### 2.3. Flow Cytometric Analysis

PBMCs (2 × 10^5^ cells) were stimulated with *M. avium* bacilli and *M. intracellulare* bacilli individually for 48 h and incubated with GolgiPlug (1 μL/mL; BD Pharmingen, San Diego, CA, USA) for the final 5 h of culture. Cells were measured by flow cytometry using a fluorescence activated cell sorter (FACS) (FACSVerse, BD Biosciences, San Jose, CA, USA) and anti-CD3-APC-Cy7, anti-CD4-FITC, anti-IFN-γ-PE, anti-IL-4-PE, anti-IL-17A-APC, anti-CD25-PE, anti-Foxp3-APC, anti-T-bet-APC, anti-GATA3-APC, and anti-RORγT-APC antibodies (BD Pharmingen, San Diego, CA, USA). The PD-1, CTLA-4, and TIM-3 were also stained with anti-PD-1-PE, anti-CTLA-4-APC, and anti-TIM-3- APC (BD Biosciences, San Diego, CA, USA). Data were analyzed using BD FACSuite software (BD Biosciences, San Jose, CA, USA). A gate was set on the lymphocytes using characteristic forward scatter and side scatter parameters followed by CD3^+^CD4^+^ T cells (Supplementary Figure S1).

### 2.4. Quantification of Cytokines

The cytokine levels in the supernatant were measured by enzyme-linked immunosorbent assay (ELISA) using commercially available IFN-γ, IL-4, IL-17A, and IL-10 ELISA kits (Biolegend, San Diego, CA, USA) according to the manufacturer’s instructions.

### 2.5. Statistical Analysis

Statistical analyses were performed using GraphPad Prism software version 8.0 (GraphPad Software, La Jolla, CA, USA). Analysis of variance and Student’s *t*-test were used to analyze the data with normal distribution (Tukey’s test for comparisons). The nonparametric Mann–Whitney test was used for variables with non-normal distribution. All tests were two-sided, and a *p*-value < 0.05 was considered statistically significant.

## 3. Results

### 3.1. Baseline Characteristics

Clinical characteristics of the 91 participants (71 MAC-PD cases and 20 control individuals) are shown in Table 1. The mean age was 60.6 years (±9.6), and 37 (52.1%) were female. Many MAC-PD cases had nodular bronchiectatic disease (*n* = 41, 57.7%). No patients were infected with HIV. The most common comorbidity was previous pulmonary tuberculosis (*n* = 27, 38.0%), followed by chronic airway disease (*n* = 12, 16.9%) and diabetes (*n* = 7, 9.9%).

### 3.2. Frequencies of Circulating CD4^+^ T Cell Subpopulations in MAC-PD Patients

**Figure 1:**
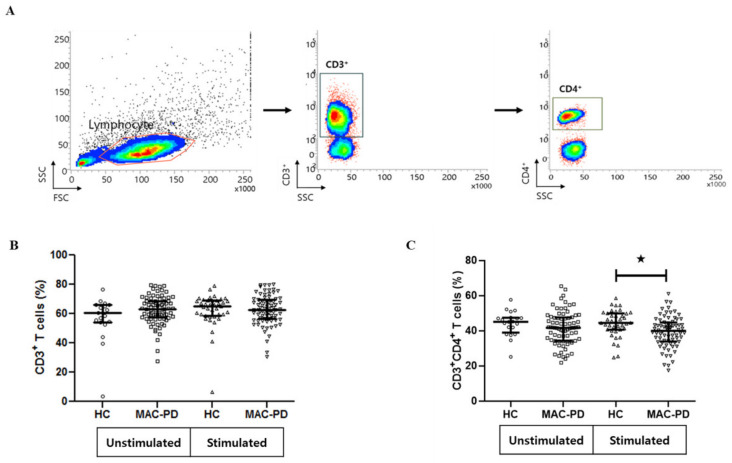
Frequency of CD3^+^ and CD3^+^CD4^+^ T cells in peripheral blood mononuclear cells (PBMCs) from MAC-PD. The proportion of CD3^+^ and CD3^+^CD4^+^ T cells in MAC-stimulated cultures. MAC-PD (*n* =71), and HC (*n* = 20). (**A**) The gating strategy for flow cytometric analysis; (**B**) comparison of proportion of CD3^+^ T cells between MAC-PD patients and control individuals (unstimulated and stimulated); (**C**) comparison of proportion of CD3^+^CD4^+^ T cells between MAC-PD patients and control individuals (unstimulated and stimulated). Each symbol represents one single individual. Horizontal bars denote mean levels in each group. Statistical comparisons were performed using a Student’ *t* test. ^★^indicate statistical significance (*p* < 0.05). MAC-PD, *Mycobacterium avium complex* pulmonary disease; MAC, *Mycobacterium avium complex;* HC, Healthy controls.

**Figure 2:**
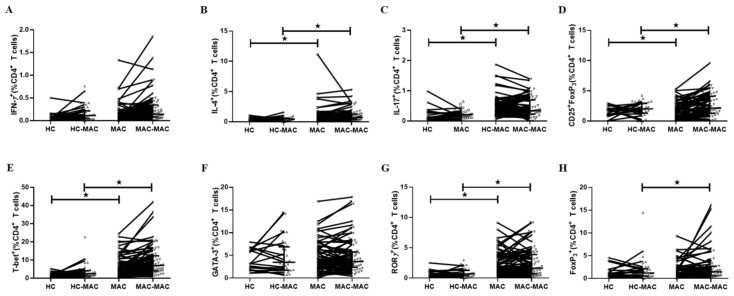
T cell cytokine profiles in MAC-PD patients. Representative flow cytometry plots of the expression of (**A**) IFN-γ, (**B**) IL-4, (**C**) IL-17, (**D**) CD25^+^Foxp3^+^ T cells, (**E**) T-bet, (**F**) GATA3, (**G**) RORγ, and (**H**) Foxp3 after simulation with MAC bacilli in one individual from each group. The frequency of each cytokine-producing cells expressed as a percentage of the total CD3^+^CD4^+^ T cell population is indicated. Bar represents the median. Symbols shown below the horizontal dashed line correspond to non-responders. Statistical comparisons were performed using a Mann–Whitney test. ^★^indicate statistical significance (*p* < 0.05). MAC-PD, *Mycobacterium avium complex* pulmonary disease; MAC, *Mycobacterium avium complex;* HC, Healthy controls; HC-MAC, MAC-stimulated in healthy controls; MAC-MAC, MAC-stimulated in MAC-PD

To characterize T cell immune phenotype to MAC, we stimulated PBMCs with heat-killed *M. avium* bacilli and *M. intracellulare* bacilli individually and evaluated the proportion of CD3^+^CD4^+^ T cells subpopulations in patients with MAC-PD patients and healthy controls. The gating strategy for flow cytometric analysis is shown in Figure 1A and Figure S1. Flow cytometric analysis indicated that the proportion of CD3^+^ T cells was similar in patients with MAC-PD (62.7%, 6490 ± 778.8 cell/μL) and control individuals(56.7%, 5742 ± 1221 cell/μL), both in cultures with and without stimulation (Figure 1B).The patients with MAC-PD had an average proportion of 41.7% CD3^+^CD4^+^ T lymphocytes, similar to the controls (44.6%). However, in MAC bacilli-stimulated PBMCs, the frequency of CD3^+^CD4^+^ T cells was lower in MAC-PD (mean 39.6%) compared with healthy controls (mean 44.4%) (Figure 1C). Within CD3^+^CD4^+^ T lymphocytes, the percentage of Th1 cells, defined as lymphocytes positive for both CD4 and IFN-γ, was comparable between the two groups (median 21.1% vs. median 18.0%, *p* = 0.686, 1800 ± 1118.4 cell/μL vs. 1037.5 ± 861.1 cell/μL), while the percentage of Th2 cells, defined as those positive for both CD4 and IL-4, was higher in the MAC-PD group than in healthy controls (median 0.83% vs. median 0.51%, *p* = 0.041, 1009.8 ± 1706.2 cell/μL vs. 766.2 ± 336.4 cell/μL). For CD4+ T cells, MAC stimulation reduced the population of IL-4 in the MAC-PD. (Figure 2A–B). Differentiation of CD4^+^ T cells into Th1 and Th2 cells is regulated by T-bet (T-box protein expressed in T cells, also called TBX21) and GATA binding protein 3 (GATA3), respectively. T-bet is a master Th1 transcription factor that control the expression of the Th1 cytokine, IFN-γ [27]. The percentage of CD4^+^T-bet^+^ cells was higher in patients with MAC-PD than control individuals, both in cultures with or without stimulation. GATA3 plays a central role in Th2 cytokine, IL-4 [28]. The proportion of CD4^+^GATA3^+^ cells was similar in patients with MAC-PD and control individuals, both in cultures with and without stimulation (Figure 2E–F).

With regard to Th17 cells, defined as those positive for both CD4 and IL-17A, a higher percentage was observed in patients with MAC-PD than in the controls (median 1.26% vs. median 0.77%, *p* = 0.011, 1466.7 ± 1090 cell/μL vs. 2619.8 ± 9334 cell/μL). The proportion of CD4^+^IL-17^+^ cells was significantly lower in MAC-stimulated MAC-PD cells compared with MAC-stimulated control cells (Figure 2C). The proportion of CD4^+^RORγT^+^ cells was higher in patients with MAC-PD than in control individuals, although it was similar in cultured cells from MAC-PD (median 2.2%, 348.3 ± 336 cell/μL) and control individuals (median 0.7%, 130.3 ± 92.8 cell/μL), with and without stimulation (Figure 2G). In addition, the percentage of CD25^+^ and Foxp3 in CD4^+^ T lymphocytes was higher in the MAC-PD group compared with the control group, with and without stimulation (median 6.31% vs. median 3.5%, *p* = 0.012, 1381.5 ± 810.2 cell/μL vs. 545.8 ± 396.2 cell/μL) (Figure 2D). 

As a result, in PBMCs stimulated with MAC, we found a significant increase in the frequency of CD4^+^IL-4^+^ (Figure 2B) and CD4^+^CD25^+^Foxp3^+^ Tregs (Figure 2D) in patients with MAC-PD compared with that in the control group.

### 3.3. T Cell Cytokine Production in MAC-PD 

**Figure 3:**
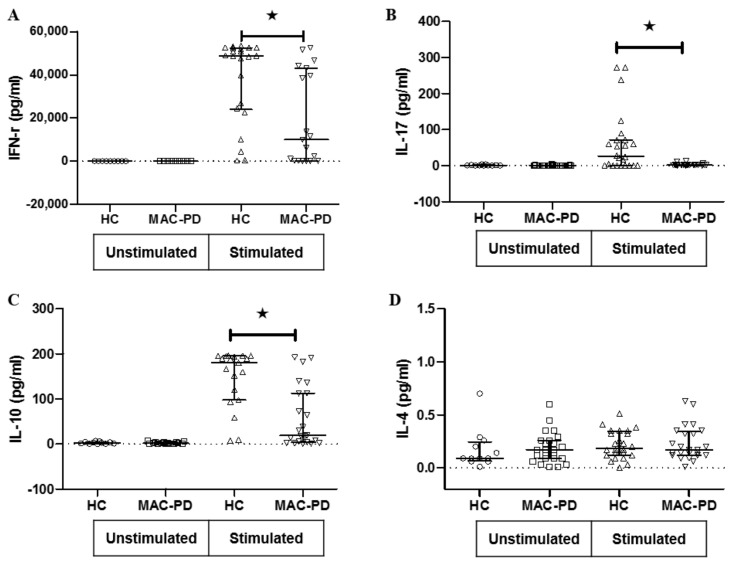
Cytokine stimulation and response for peripheral blood mononuclear cells in MAC-PD patients. Cytokine responses were measured after treating peripheral blood mononuclear cells for 48 h with heat-killed MAC bacilli (a multiplicity of infection [MOI]:100) in MAC-PD and HC. Values for (**A**) IFN-γ, (**B**) IL-17, (**C**) IL-10, and (**D**) IL-4 are presented as dot plots with crossed lines of mean values and were analyzed using a Student’ *t*-test. ^★^indicate statistical significance (*p* < 0.05). MAC-PD, *Mycobacterium avium complex* pulmonary disease; MAC, *Mycobacterium avium complex;* HC, Healthy controls.

Next, we investigated secretion of cytokines related to T cell subsets in the supernatants from MAC bacilli (MOI = 100) stimulated and unstimulated cultures. IFN-γ production in response to MAC stimulation was significantly decreased in cultures from patients with MAC-PD (mean 19.0 ± 21.1 ng/mL) compared with that from control participants (mean 38.2 ± 19.1 ng/mL) (Figure 3A). Similarly, IL-17A production in response to MAC stimulation was significantly decreased in cultures from patients with MAC-PD (mean 2.6 ± 3.0 ng/mL) than from control individuals (mean 61.0 ± 84.0 pg/mL) (Figure 3B). The IL-10 production in response to stimulation by MAC was significantly decreased in patients with MAC-PD (mean 69.9 ± 68.6 ng/mL) compared with controls (mean 145.4 ± 65.4 ng/mL) (Figure 3C). The concentration of IL-4 in response to stimulation was not significantly different between MAC-PD patients (mean 0.2 ± 0.2 pg/mL) and controls (mean 0.2 ± 0.1 pg/mL) (Figure 3D).

### 3.4. Expression of Multiple Inhibitory Receptors in Circulating CD4^+^ T Cells in MAC-PD

**Figure 4:**
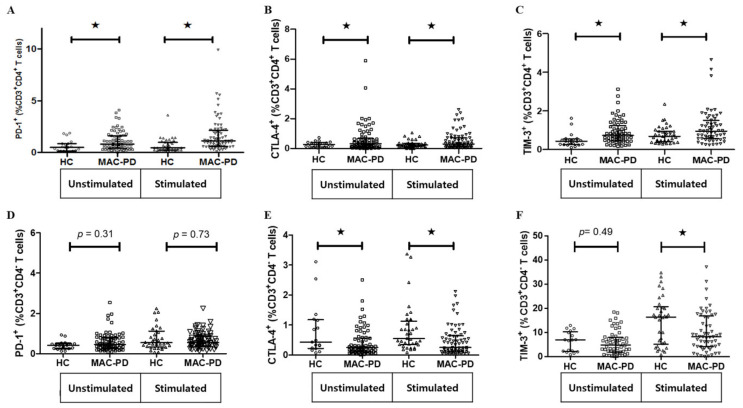
Expression of PD-1, CTLA-4, and TIM-3 after ex vivo stimulation of PBMCs. Data are shown as scatter plots of the percentage of (**A**–**C**) the CD3^+^CD4^+^ T cell and (**D**–**F**) the CD3^+^CD4^-^ T cell population expressing PD-1, CTLA-4, and TIM-3 with median for MAC-PD compared with HC. Statistical comparisons were performed using a Mann–Whitney test. ^★^indicate statistical significance (*p* < 0.05). MAC-PD, *Mycobacterium avium complex* pulmonary disease; MAC, *Mycobacterium avium complex;* HC, Healthy controls.

It is well documented that T cell dysfunction is mediated by several immune inhibitors including PD-1, CTLA-4, and TIM-3 [17]. To further investigate whether immune inhibitors modulated on CD4^+^ T cells from MAC-PD patients, we observed the expression of PD-1, CTLA-4 and TIM-3 expression on CD4^+^ T cells obtained from MAC-PD patients compared with control individuals. Intriguingly, the expression of all investigated immune checkpoint receptors, PD-1, CTLA-4 and TIM-3 was significantly higher on CD4^+^ T cells from MAC-PD patients compared with those from controls after stimulation with MAC (Figure 4A–C). However, expressions of CTLA-4 and TIM-3 were significantly lower on CD3^+^CD4^-^ T cells from MAC-PD patients compared with those from controls after stimulation with MAC (Figure 4D–F).

## 4. Discussion

In the current study, we observed the immunological features of circulating CD4^+^ T lymphocytes in patients with MAC-PD and found a lower frequency of Th17 cells, but a higher frequency of Th2 cells and Tregs compared to those in control individuals. In addition, our study revealed MAC-induced CD4^+^ T cell dysfunction and represented significantly higher populations of PD-1^+^CD4^+^cells, CTLA-4^+^CD4^+^cells, and TIM-3^+^CD4^+^ T cells in MAC-PD patients compared with healthy controls. We also showed that the expressions of PD-1, CTLA-4, and TIM-3 were significantly induced upon MAC stimulation in PBMCs of MAC-PD patients compared with those of controls.

IFN-γ plays a pivotal role in immune defense against mycobacteria [29]. In a recent NTM study, PBMCs of NTM patients cultured with anti-CD3, phytohaemagglutinin (PHA), or MAC showed a decrease in Th1 cytokines compared with that from healthy controls. Vankayalapati et al. found that PBMCs from patients with active pulmonary MAC produced lower IFN-γ, IL-12, and TNF-α than *M. avium* sensitive–responsive control individuals [30]. We, and others, report a marked decrease in IFN-γ secretion in response to stimulation with PHA and diminished production of IFN-γ and TNF-α in MAC bacilli-stimulated PBMCs from patients with MAC-PD [25,31]. However, contradictory results have also been reported [11]. In the present study, IFN-γ production decreased in response to MAC bacilli in PBMCs from MAC-PD patients, but no difference was observed in the frequencies of IFN-γ^+^ CD4^+^ T cells in NTM-PD. Therefore, although Th1 lymphocytes are important in the immune response to mycobacterial infection, IFN-γ alone might be insufficient for complete eradication of the bacteria, suggesting roles for other cytokines in the immune defense response against mycobacteria.

Successful host defense against mycobacteria with clearance/control of mycobacterial infection requires an effective Th1 and, to a lesser extent, functioning Th17 immunological response rather than a Th2-type response [32,33]. However, chronic disequilibrium between the different divisions of the adaptive immune system may lead to pathology and susceptibility to infection [34]. In a previous cohort study of patients infected with *M. tuberculosis*, peripheral blood eosinophil count and serum IgE levels in patients with MAC-PD were higher than those in patients with pulmonary TB or other species of NTM [35]. We observed a similar phenomenon, wherein the frequency of IL-4^+^ CD4^+^ T cells was elevated in MAC-stimulated PBMCs from patients compared with that from control individuals, although there was no change in IL-4 production. The discrepancy between this study and others may be explained by the possibility that MAC may have developed defensive mechanisms to skew immune responses towards a Th2-type response, thereby decreasing the ability of the immune system to clear the mycobacteria.

In the present study, we observed elevated levels of IL-17 in MAC-PD patients. In addition, The IL-17 level from lymphocytes was higher after MAC bacilli stimulation both of MAC-PD patients and healthy controls, but the IL-17 level expressed from lymphocytes activated in MAC-stimulated PBMCs was lower in patients with MAC-PD than that in healthy controls. This result is consistent with a previous study conducted on *M. avium* in macrophages [25]. A recent study showed increased IL-17 and IL-23 gene expression in the lungs of patients with active TB [36], while another reported that MAC lung disease was associated with defects or biases in Th1 and Th17 immunity [11,37]. Thus, the attenuated IL-17 response might contribute to host vulnerability or pathogen evasion in MAC-PD via impairment of neutrophil recruitment and granulopoiesis.

Tregs play a role in the immunosuppression observed in chronic infectious diseases [38], suggesting that Tregs might contribute to impaired specific MAC-induced T cell responses. Antigen-stimulated blood cells from NTM-PD patients showed an elevated Treg population compared with that from control individuals [39]. Furthermore, Tregs were increased in the peripheral blood of cystic fibrosis NTM patients compared with that of controls [24]. Our study demonstrates that Tregs were increased in MAC-stimulated PBMCs of MAC-PD patients compared with that of control individuals, suggesting that the elevated levels of Tregs might suppress T-cell responses, thereby protecting MAC and playing a pathogenic role in MAC-PD.

Recent studies demonstrated that PD-1 regulates T cell activation, peripheral tolerance and autoimmunity, principally as an inhibitory molecule [40,41]. Several studies have demonstrated that the PD-1 signaling pathway is activated during persistent infection with various microorganisms and contributes to impairment of protective immunity [22]. Few studies have addressed the effects of PD-1 on T lymphocytes in NTM infection. It was recently shown that in vitro blockade of PD-1 signaling enhanced MAC-specific IFN-γ production by T cells [25] and NK T cells of PTB patients [42], indicating that this inhibitory pathway also affects T cell functions during mycobacterial infection. Elevated CTLA-4 was also observed on CD4^+^ T cells in NTM infection [24]. Consistent with these reports, we found that PD-1^+^CD4^+^ T cells and CTLA-4^+^CD4^+^ T cells were more prevalent in patients with MAC-PD compared with that in control participants. In addition to these molecules, there is a variety of other immune checkpoints with unknown function in relation to mycobacteria. Tim-3 is a membrane protein expressed at late stages of interferon-gamma secreting CD4^+^ T helper type 1 (Th1) cell differentiation [43]. T-bet binds directly to the Tim-3 promoter in CD4^+^ Th1 cells [44]. Among inhibitory factors, TIM-3 has not been studied with respect to MAC-PD. Our data indicate, for the first time, that TIM-3 is elevated in CD4^+^ T cells after MAC infection, further indicating the degree of immunosuppression in MAC-PD.

T cell exhaustion is common during chronic infections. Exhausted CD4^+^ T cells display poor production of effector cytokines (TNF and IFN-γ) and express high levels of PD-1 [45]. It has been suggested that CD4^+^ T cells increased expression of mRNA encoding several transcription factors including PR domain zinc-finger protein 1, nuclear factor of activated T cell, and T-bet have been implicated in the development of different T cell subsets in chronic infection [46]. We observed a decrease in effector cytokine production and the high populations of transcription factors related T cells subset in MAC-stimulated PBMCs in MAC-PD. The low IFN-γ and IL-17 production by MAC stimulated- CD4^+^ T cells probably explained by exhausted CD4^+^ Th1 cells to induce the binding of T-bet to the Tim-3 promoter. T-bet has been reported to be a transcription factor for regulating Tim-3 promoter during chronic infection [44]. T-bet enhances Tim-3 expression via a c-Jun N-terminal kinases pathway, leading to dampened monocyte/macrophages function during hepatitis C virus infection [47].

This study had several limitations. First, the patients and controls were not age-matched. The average age of the patients with MAC-PD was higher than that of the controls, possibly leading to some bias, although we found similar results in age-matched subgroups. Second, an MOI of 100 is high, warranting caution; thus, further validation studies using viable bacilli are needed. Third, we could not identify the intracellular mechanism by which MAC induces the expression of PD-1, CTLA-4, and TIM-3. Finally, we did not perform lymphocyte proliferation assays or intracellular cytokine staining to evaluate lymphocyte function. 

In summary, our study provides evidence for CD4^+^ T cell dysfunction in patients with MAC-PD. The population of CD4^+^ and CD4^+^IL-17^+^ T cells was decreased in MAC-induced PBMCs from patients with MAC-PD, while that of CD4^+^IL-4^+^ and CD4^+^CD25^+^Foxp3^+^T cells was increased in MAC-induced PBMCs from patients with MAC-PD. An increasing population of PD-1, CTLA-4, and TIM-3 might be responsible for Th1, Th2 and Th17 cells in MAC-PD. Our findings suggest a complex immune response in MAC-PD patients and targeted interventions against the inhibitor pathways may help restore local and systemic immunity in these patients.

## Figures and Tables

**Table 1:** Clinical characteristics of the study population.

	MAC-PD	HC
	(*n* = 71)	(*n* = 20)
MAC species		
*M. avium*	30 (42.3)	
*M. intracellulare*	41 (57.7)	
Age, years	60.6 ± 9.6	46.3 ± 7.9
Female	37 (52.1)	16 (22.5)
Radiologic feature		
Nodular bronchiectatic form	41 (57.7)	
Fibro-cavitary form	30 (42.3)	
Comorbidities		
Previous pulmonary tuberculosis	27 (38.0)	
Chronic airway disease	12 (16.9)	
Chronic pulmonary aspergillosis	2 (2.8)	
Idiopathic pulmonary fibrosis	1 (1.4)	
Diabetes	7 (9.9)	
Chronic kidney disease	1 (1.4)	
Chronic liver disease	2 (2.8)	
Rheumatic disease	2 (2.8)	

Data are presented as n (%) or mean with standard deviation. MAC-PD, *Mycobacterium avium complex* pulmonary disease; HC, Healthy controls.

## Supplementary Materials

The following are available online at https://www.mdpi.com/2077-0383/9/5/1331/s1, Figure S1: Overview of flow cytometry gating strategy. FSC, forward scatter; SSC, side scatter.
