# Supplementary figures and images for: Characteristics of Circulating CD4+ T Cell Subsets in Patients with Mycobacterium avium Complex Pulmonary Disease
Source: J Clin Med. 2020 May 3;9(5):1331. doi: 10.3390/jcm9051331 (PMC7290757; doi:10.3390/jcm9051331)

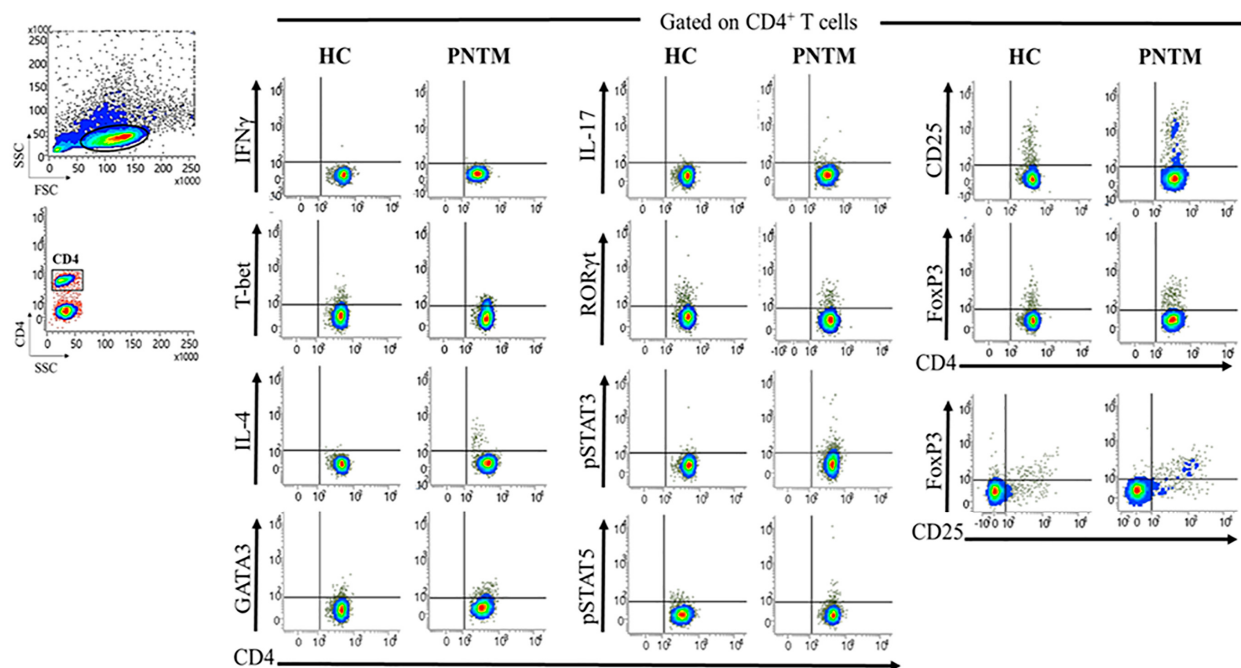

**Figure S1.** Overview of flow cytometry gating strategy. FSC, forward scatter; SSC, side scatter.

Supplement: Supplementary file 1 [file jcm-09-01331-s001.pdf]
